# Deciphering *KRAS* and *NRAS* mutated clone dynamics in MLL-AF4 paediatric leukaemia by ultra deep sequencing analysis

**DOI:** 10.1038/srep34449

**Published:** 2016-10-04

**Authors:** Luca Trentin, Silvia Bresolin, Emanuela Giarin, Michela Bardini, Valentina Serafin, Benedetta Accordi, Franco Fais, Claudya Tenca, Paola De Lorenzo, Maria Grazia Valsecchi, Giovanni Cazzaniga, Geertruy te Kronnie, Giuseppe Basso

**Affiliations:** 1 Department of Woman and Child Health, University of Padua, Via Giustiniani 3, 35128 Padua, Italy; 2 Centro Ricerca Tettamanti, Department of Paediatrics, University of Milano Bicocca, Fondazione MBBM, Monza, Italy; 3 Department of Experimental Medicine, University of Genoa, Genoa, Italy; 4 IRCCS AOU San Martino - IST Istituto Nazionale per la Ricerca sul Cancro, Genova, Italy; 5 Biostatistics Center, School of Medicine and Surgery, University of Milano-Bicocca, Monza, Italy

## Abstract

To induce and sustain the leukaemogenic process, MLL-AF4+ leukaemia seems to require very few genetic alterations in addition to the fusion gene itself. Studies of infant and paediatric patients with MLL-AF4+ B cell precursor acute lymphoblastic leukaemia (BCP-ALL) have reported mutations in *KRAS* and *NRAS* with incidences ranging from 25 to 50%. Whereas previous studies employed Sanger sequencing, here we used next generation amplicon deep sequencing for in depth evaluation of *RAS* mutations in 36 paediatric patients at diagnosis of MLL-AF4+ leukaemia. *RAS* mutations including those in small sub-clones were detected in 63.9% of patients. Furthermore, the mutational analysis of 17 paired samples at diagnosis and relapse revealed complex RAS clone dynamics and showed that the mutated clones present at relapse were almost all originated from clones that were already detectable at diagnosis and survived to the initial therapy. Finally, we showed that mutated patients were indeed characterized by a *RAS* related signature at both transcriptional and protein levels and that the targeting of the RAS pathway could be of beneficial for treatment of MLL-AF4+ BCP-ALL clones carrying somatic *RAS* mutations.

Acute lymphoblastic leukaemia (ALL) with the *MLL-AF4* fusion gene represents a very aggressive leukaemia subtype associated with poor prognosis^1^,^2^,^3^. The translocation involves the N-terminus of *MLL* (chromosome 11q23) that fuses in frame with the C-terminus of *AF4* (chromosome 4q23) and leads to the balanced *MLL-AF4* and *AF4-MLL* fusion genes^4^. Several studies investigated the leukaemogenic properties of both fusion genes *in vitro* and *in vivo* providing important insights into the pathogenesis of MLL-related leukaemia^3^,^5^,^6^. Nevertheless, *in vivo* studies provided discrepant results and mouse models resembling the features of the human MLL leukaemia are still missing^3^,^7^,^8^,^9^. In particular, the relative long disease latency^7^,^8^,^9^,^10^,^11^ in mice models argued against the concept that the MLL fusion gene alone is sufficient for full transformation and suggested that cooperating events are required to induce ALL. Whole genome sequencing^12^,^13^ as well as SNP analysis^14^,^15^ of *MLL-AF4* positive patients reported very few additional genomic alterations suggesting that the *MLL* fusion *per se* predisposes cells to malignant transformation. However, patients with MLL rearranged leukaemia display high *FLT3* expression^16^,^17^ and *in vivo* data showed that mutations in *FLT3* accelerate MLL-induced leukaemogenesis^18^. Similarly, a transgenic mouse model simultaneously expressing *MLL-AF4* and an activated *KRAS* mutated gene showed a shortened leukaemia latency^11^, which prompted for renewed *KRAS* and *NRAS* mutation screenings in *MLL* rearranged and wild type infant and paediatric patients.

Recently, several works showed the potency of next generation sequencing in revealing precise frequency of mutations even at very low frequencies^19^,^20^,^21^. In this study, we deeply investigated the *RAS* mutational status in infant and paediatric MLL-AF4+ patients and reported the results of ultra sensitive deep mutation sequencing of *KRAS* and *NRAS* hot spot regions in MLL-AF4+ paediatric patients at diagnosis. Moreover, to trace *RAS* mutations during the disease course we sequenced paired diagnosis and relapse samples. Furthermore, we studied the expression and proteomic profiles of *RAS* mutated (RAS^mut^) patients looking for an activated RAS-related signature and we also evaluated the possibility to target the *RAS* pathway in MLL-AF4+ RAS^mut^ samples.

## Results

### Detection of *KRAS* and *NRAS* variants at diagnosis

**Figure 1:**
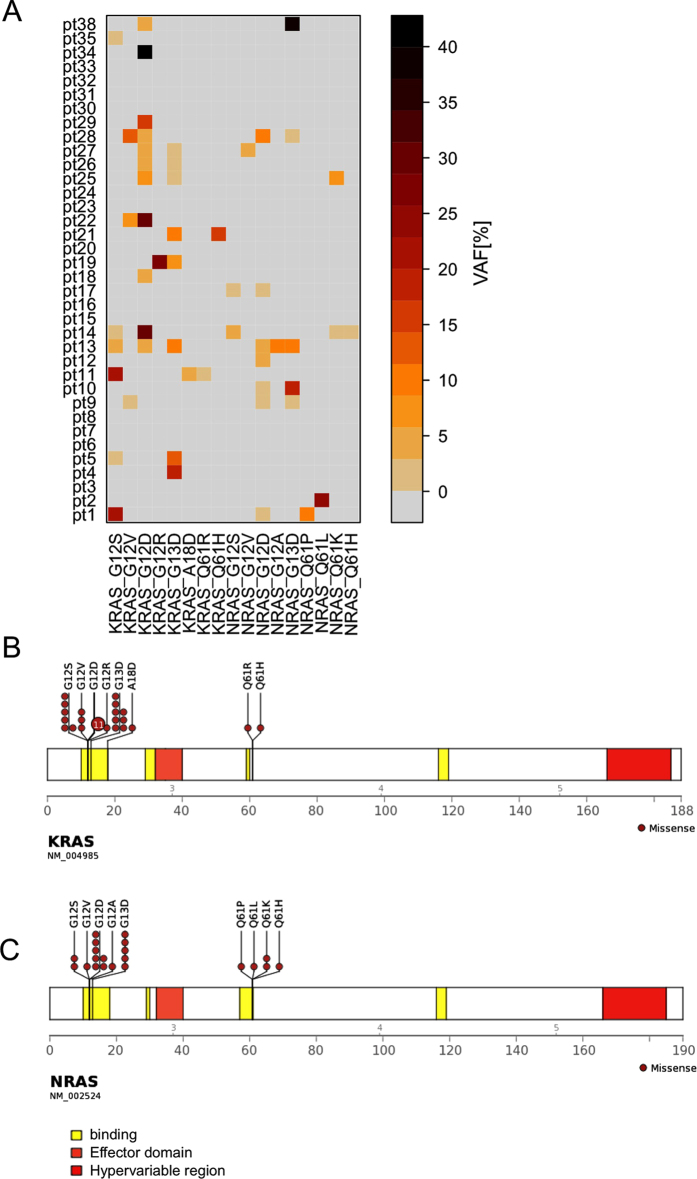
*RAS* mutational profiles of infant and non-infant MLL-AF4 positive patients. **(A)** Distributions of *KRAS* and *NRAS* mutations in the 36 analysed patients at diagnosis according to ultra deep sequencing analysis. The variant allele frequency (VAF) is reported as percentage using a colour code scale. **(B)** Schematic diagrams of all the identified mutations in *KRAS* and *NRAS* with VAF >1% with respect to proteins functional domains.

Mutations with variant allele frequency (VAF) ≥1%^20^,^22^ were considered for analysis according to the calculated sensitivity threshold (Supplementary Figure 1). In total, we identified 23 mutated (63.9%) and 13 not mutated (36.1%) patients at diagnosis, Fig. 1A, Supplementary Tables 1 and 2. In particular, 16 out of 23 patients (69.6%) carried mutations in more than one clone, irrespective of the *RAS* mutated genes, whereas only 7 out of 23 (30.4%) carried mutations in one single clone. Moreover, clones harbouring different mutations at exactly the same genomic hotspot were also detected (ex. patients 13, 14, 22).

Interestingly, 8 out of 23 patients (34.8%) simultaneously carried clones with both *KRAS* and *NRAS* mutations; on the other hand, 9 patients presented mutations only in *KRAS* whereas the remaining 6 patients (26.1%) harboured *NRAS* mutations only.

Overall, 29 mutations in *KRAS* exon 2 and 2 mutations in *KRAS* exon 3 were detected; only one *KRAS* mutation (A18D) and one silent *KRAS* mutation (A66A) were identified in a non hot spot region with a VAF ≥ 1%. The *KRAS* and *NRAS* mutations most frequently identified were G12D (11 clones) and G12D (7 clones) respectively, Fig. 1B,C.

### RAS mutations and patients’ outcome

Clinical and outcome data were available for 34 out of 36 patients, 22 infants (aged 1 year or less at diagnosis) and 12 paediatric patients. Patients’ characteristics are described in Table 1. *RAS* mutations were significantly more frequent in infants as compared to older children: RAS^mut^ were 17/22 (77%) among infants, while only 4/12 (33%) in the paediatric cohort (p-value = 0.0248). No other significant association was observed between occurrence of any *RAS* mutation and relevant characteristics at diagnosis, i.e. sex, WBC count and PDN response. Types of events and outcome are described in Supplementary Table 3. All but one patient reached complete remission at the end of the induction phase. Most events were relapses and the 4-year CIR was markedly different according to RAS status, being 71.4 (SE 10.5) and 46.2 (SE 14.8) for RAS^mut^ and RAS^wt^ patients, respectively (p-value = 0.14). In agreement with previous data^23^, patients with a *RAS* mutation had a worse outcome than patients without RAS mutations, with a 4-year EFS of 14.3 (SE 10.9) vs. 38.5 (SE 13.8), although the difference was not statistically significant (p-value = 0.16), Supplementary Fig. 2.

### RAS mutations in paired diagnosis-relapse patients’ samples

To assess the development of *RAS* mutations along the disease course we analysed seventeen paired samples at diagnosis and relapse. Fourteen of these 17 paired samples (82.3%) presented *RAS* mutations at diagnosis; 3 patients did not show a *RAS* mutation at diagnosis and, similarly, did not acquire a *RAS* mutation at relapse. Seven of the fourteen mutated diagnostic samples (50%) still harboured a *RAS* mutation at relapse and 6/7 (85.7%) relapsed mutated patients presented at least one of the mutated clones detected at diagnosis also at relapse. Additionally, *de novo* mutated clones were also detected at relapse in patient 18 (3 clones) and patient 22 (1 clone), Supplementary Figure 3, Supplementary Table 1 and Supplementary Table 4. Analysing the data with respect to VAF, we observed that 4/5 patients (80%) (i.e. patient 9, 12, 26, 35) with a total VAF (i.e. VAF index) <10% at diagnosis lost their primary *RAS* mutated clones at relapse and only patient 18 presented four mutated clones at relapse, one from the diagnosis and three acquired *de novo* at relapse. On the contrary, 8/9 (88.9%) patients (i.e. patients 4, 10, 21, 22, 25, 27, 28, 29) with VAF index at diagnosis >10% lost some of their initial clones but, at the same time, 5/9 (55.5%) patients (i.e. patients 10, 25, 27, 28, 34) maintained clones already present at diagnosis or even acquired a *de novo* mutation (i.e. patient 22).

**Figure 2:**
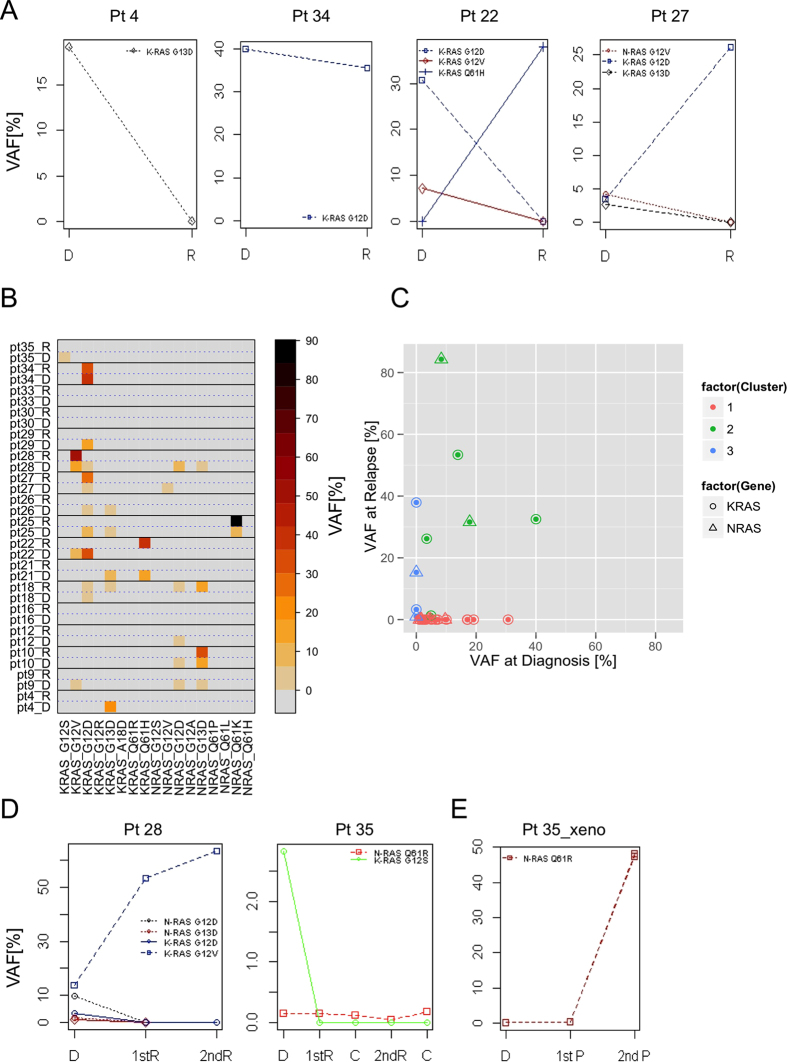
RAS clones dynamics over time. **(A)** Plots exemplifying the four different behaviours of the mutated clones identified in paired samples at diagnosis (D) and relapse (R). **(B)** Distributions of *KRAS* and *NRAS* mutations in the 17 analysed patients at diagnosis (D) and relapse (R) according to ultra deep sequencing analysis. The variant allele frequency (VAF) is reported as percentage using a colour code scale. Straight lines separate individual patients and dotted lines divide data measured at diagnosis and relapse. **(C)** The variant allele frequency (VAF) of mutated clones is reported as percentage in matched samples at diagnosis and relapse allowing the identification of three major clusters. Cluster 1: mutations present at diagnosis only; cluster 2: mutations with a constant and/or increasing VAF over time; cluster 3: *de novo* mutations at relapse. Circle: *KRAS* mutation; triangle: *NRAS* mutation. **(D)** RAS mutated clones in first relapse (1st R), second (2nd R) relapse and in available control time points (**C**) in patient (Pt) 28 and patient (Pt) 35. **(E)** Detection of RAS mutated clones in DNA isolated from bone marrow cells of xenotransplanted NOD/SCID mice (N = 2) using patient 35 BM cells at diagnosis. 1st P: primary passage; 2nd P: secondary passage.

In general, four distinct behaviours of RAS^mut^ clones were identified, Fig. 2A: (i) clones at diagnosis disappearing at relapse, (ii) clones at diagnosis stably maintained at relapse, (iii) mutated clones not present at diagnosis appearing *de novo* at relapse, (iv) mutated clones at diagnosis evolving at relapse. The distribution of RAS mutated clones in paired diagnosis and relapse samples is shown in Fig. 2B,C and could be grouped in 3 major clusters: (i) mutations present at diagnosis and no longer detected at relapse (cluster 1), (ii) mutations stable from diagnosis to relapse or expanding at relapse (cluster 2) and (iii) *de novo* mutations at relapse (clusters 3).

Interestingly, the expansion of a clone at relapse was not related to the mutation load at diagnosis: e.g. in two patients (i.e. patients 18 and 22) the presence of the mutated clone at relapse was independent of the frequency of the variants of the clones at diagnosis, suggesting a positive clonal selection/survival over time. A similar selection process was seen in other three patients (i.e. patients 25, 27, 28) presenting at relapse only one of the clones that had been detected at diagnosis even if the clones at diagnosis had a similar mutation load.

In two cases (i.e. patient 28 and patient 35) we analysed also the DNA at second relapse (patient 28) and at two control time points (patient 35), Fig. 2D. In patient 28, the mutated clone identified at diagnosis and at primary relapse was maintained also at second relapse. Conversely, in patient 35, the *KRAS* G12S clone detected at diagnosis disappeared at subsequent time points.

The selection process acting on RAS^mut^ leukaemia clones that we postulated based on the analysis of patient samples at different time points was also confirmed in NOD/SCID mice (n = 2 mice/passage) serially transplanted with the BM cells of patient 35. The clone harbouring the *KRAS* G12S mutation present at diagnosis was negatively selected and not detectable in engrafted mice whereas a major clone carrying the *NRAS* Q61R mutation resulted to be the only one present in leukaemia cells isolated from leukaemia bearing mice at secondary passage, Fig. 2E. Of note, backtracking the *NRAS* Q61R clone in patient 35 specimens, we observed that this clone was present at all time points but with a VAF below the 1% cut-off -threshold, Fig. 2D.

### Detection of an activated RAS-related profile

**Figure 3:**
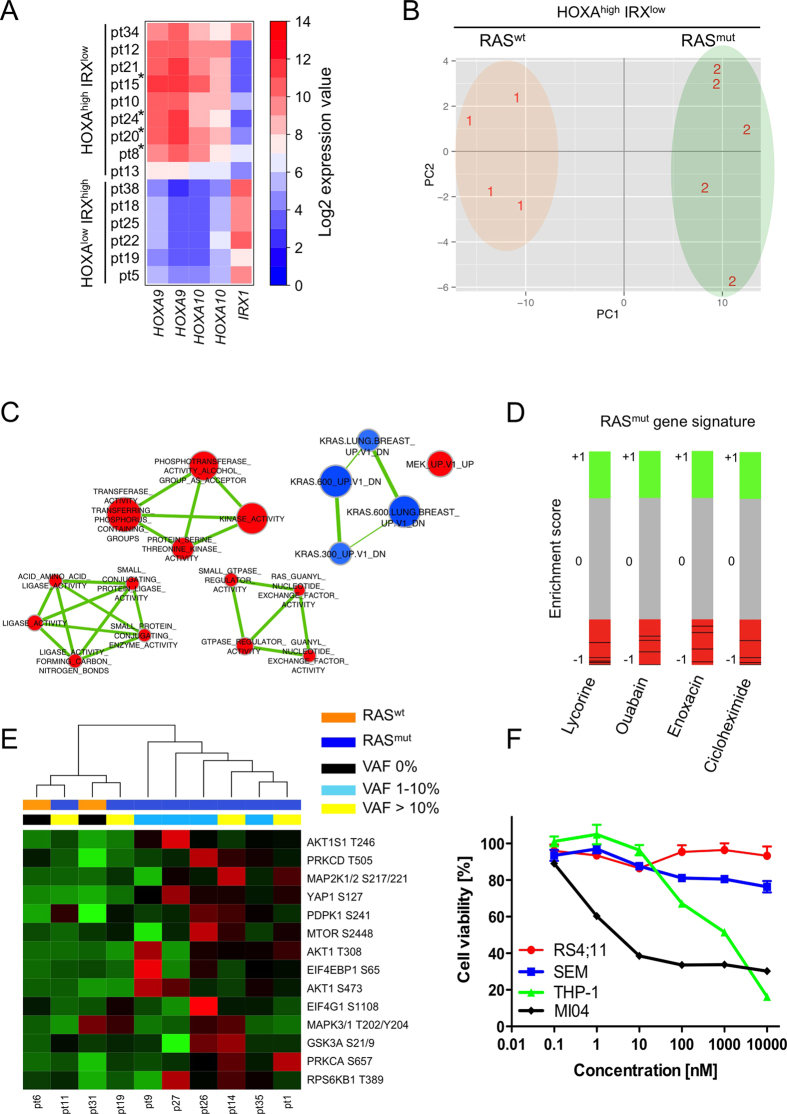
Detection of a RAS related signature in RAS^mut^ patients. **(A)** *HOXA9* (209905_at and 214651_s_at), *HOXA10* (213150_at and 213147_at) and *IRX1* (230472_at) expression levels in 15 MLL-AF4 positive patients at diagnosis. *: RAS wild type (RAS^wt^) patients. **(B)** Principal component analysis (PCA) of HOXA^high^IRX^low^/RAS^mut^ and HOXA^high^IRX^low^/RAS^wt^ patients. The samples were projected into a 2-dimensional space (PC1 and PC2) consisting of the differentially expressed genes (FDR q-val < 0.05) between HOXA^high^IRX^low^/RAS^mut^ (N = 5; label: 2) and HOXA^high^IRX^low^/RAS^wt^ (N = 4; label: 1) patients. Samples with similar characteristics will cluster together. **(C)** Enrichment map visualization of significant (FDR q-val < 0.05) enriched gene sets comparing HOXA^high^IRX^low^/RAS^mut^ and HOXA^high^IRX^low^/RAS^wt^ patients. Gene sets are depicted as circles (nodes) with edges indicating overlap between nodes. Node size is proportional to gene set size and the edges thickness shows the degree of overlap among the nodes. Red and blue colours indicate positive and negative enrichment in HOXA^high^IRX^low^/RAS^mut^ samples, respectively. **(D)** Bar-views according to connectivity map analysis. Each black line represents an individual treatment instance ordered according to its corresponding connectivity score (+1 and −1) with respect to the query signature based on genes differentially expressed between HOXA^high^IRX^low^/RAS^mut^ and HOXA^high^IRX^low^/RAS^wt^ patients with FDR q-val < 0.1. Instances at the bottom (connectivity score: −1) are more strongly anti-correlated with the query signature indicating the possibility to revert the RAS^mut^ phenotype. **(E)** The hierarchical clustering analysis of RAS^mut^ (N = 8) and RAS^wt^ (N = 2) patients samples according to proteins levels measured by RPPA reveals two clusters. One cluster contains all but 2 RAS^mut^ patients and the second one contains the 2 RAS^wt^ and the remaining 2 RAS^mut^ patients. Rows represent each analysed protein and columns represent patients. The red and green colours reflect high and low expression level, respectively. **(F)** MTT cell viability assay in the MLL-AF4+ RAS^mut^ cell line MI04 and in two RAS^wt^ MLL-AF4+ cell lines (i.e. RS4; 11 and SEM) treated for 72 h with increasing concentration of PD0325901. The myeloid cell line THP-1 with the *NRAS* G12D and the *MLL-AF9* fusion gene was use as positive control. Data represent mean values ± s.d. of three independent experiments.

To investigate if the presence of *RAS* mutated clones was associated with a distinct leukaemia transcriptional profile, fifteen samples (n = 4 RAS^wt^ and n = 11 RAS^mut^) with available gene expression data were analysed. We first examined the expression levels of *HOXA9, HOXA10* and *IRX1* genes that were previously shown to discriminate within *MLL-AF4* leukaemia two distinct signatures^24^,^25^,^26^, Fig. 3A. Six patients with low *HOXA9, HOX10* (HOXA^low^) and high *IRX1* (IRX^high^) expression were identified; all 6 HOXA^low^ IRX^high^ patients were RAS^mut^ (no HOXA^low^ RAS^wt^ patients were available) whereas the 9 HOXA^high^ IRX^low^ patients consisted of 5 RAS^mut^ and 4 RAS^wt^ patients. Aiming (i) to identify a gene signature reflecting the effect of an activated RAS signalling and (ii) to avoid comparison of HOXA^low^ and HOXA^high^ RAS^mut^ patients versus HOXA^high^ RAS^wt^ patients only as this results in the detection of genes belonging to the HOXA^high^/HOXA^low^ signature (i.e. *HOXA5, HOXA9, HOXA10, IRX2, IRX1*, Supplementary Figure 4), we decided to exclude the 6 HOXA^low^ IRX^high^ patients from further analysis. The 9 samples with high *HOXA9, HOXA10* (HOXA^high^) and low *IRX1* levels (IRX^low^) presenting both RAS^wt^ (n = 4) and RAS^mut^ (n = 5) clones had a total VAF (VAF index) ranging from 4.69% to 40% (mean value = 26%) and were used to identify transcriptional features related to the *RAS* mutation status. We identified a clear gene signature distinguishing the HOX^high^/IRX^low^ patients according to their *RAS* mutational status, Fig. 3B and Supplementary Table 5. Furthermore, the presence of a gene expression profile consistent with *RAS* pathway activation was confirmed by enrichment modules analysis of GSEA data. Indeed, RAS^mut^ patients showed a negative enrichment for down-regulated *KRAS* related genes and a positive enrichment for up-regulated *MAP2K1*, GTP-ases, phosphorylation and ligase-related signatures, Fig. 3C. Moreover, the connectivity map analysis on the RAS^mut^ patients’ signature identified within the top 15 ranked perturbagenes 4 compounds (i.e. lycorine, ouabain, enoxacin, cicloheximide) with negative enrichment score linked to the RAS activity modulation and potentially able to revert the RAS mutated phenotype^27^,^28^,^29^,^30^, Fig. 3D and Supplementary Table 6. To confirm the presence of an active *RAS* related signature also at protein level, we performed a reverse phase protein arrays (RPPAs) analysis on 2 *RAS*^*wt*^ samples and 8 RAS^mut^ samples with a VAF index ranging from 2.83% to 37.6% (mean value = 19.2%) assessing the phosphorylation status of several RAS-downstream targets in *MLL-AF4* patients at diagnosis. The analysed molecules belong to the transcription/cell cycle regulation (MAPK3/MAPK1, MAPKAPK2), cell proliferation (PIK3CA-AKT1-MTOR) and the Ca^2+^ signalling (PRKCA and PRKCD) pathways. Moreover, we also included YAP1 that was recently shown to be stabilized by mutated RAS^31^ and to be crucial for proliferation of mutant KRAS neoplastic pancreatic cells^32^. RAS^wt^ and RAS^mut^ patients formed almost two distinct clusters in an unsupervised analysis with only two RAS^mut^ patients with VAF >10% clustering in the RAS^wt^ branch, Fig. 3E.

### Targeting RAS in MLL-AF4 leukaemia

The presence of RAS mutations at diagnosis detected in 63.9% of patients as well as gene and protein expression data supporting the presence of an active RAS-related profile in patients harbouring RAS mutations prompted us to explore the possibility of targeting the RAS pathway as a potential therapeutic approach in BCP-ALL MLL-AF4+ RAS^mut^ leukaemia. We choose to indirectly target RAS using PD0325901, a potent inhibitor of MAP2K1/2 kinase^33^ currently in phase II clinical trials^34^. The *NRAS* Q61K mutated MLL-AF4+ B cell line MI04 displayed high sensitivity at 72h post-treatment with PD0325901 even using nanomolar dosages, whereas the two RAS^wt^ MLL-AF4+ cell lines (RS4;11 and SEM) showed only a modest proliferative reduction even increasing the drug concentration. Of note, 10nM PD0325901 showed 60% reduction in growth capability of the RAS^mut^ MI04 cell line at 72h of treatment, Fig. 3F.

## Discussion

The capability of the *MLL-AF4* fusion gene to sustain leukaemogenesis without additional genetic alterations is still not completely clear. Several works showed that BCP MLL-AF4 rearranged leukaemia is characterized by a very low rate of genetic alteration^13^,^14^,^15^ and using Sanger sequencing a significant amount of data on the mutational status of *RAS* genes in infants and paediatric patients with MLL-AF4 leukaemia were previously obtained^23^,^35^,^36^,^37^. However, the percentage of *RAS* mutated patients showed a certain inconsistency among these studies ranging from 25 to 50%^23^,^35^,^36^,^37^. In the present study we analysed the mutational status of *NRAS* and *KRAS* hot spot regions in 36 *MLL-AF4* patients taking advantage of the ROCHE 454 massive parallel sequencing approach that offers a powerful tool to investigate clonal heterogeneity and detect minor mutated clones^19^,^20^,^21^. Thanks to the ultra deep sequencing, we identified *RAS* mutations in 63.9% of patients at diagnosis. This higher recurrence of *RAS* mutations measured in our samples compared to previously published data can be attributed to the sample cohort as well as to the considerable increase in sensitivity of next generation sequencing (NGS) technology^20^,^38^,^39^ compared to Sanger-based sequencing (Supplementary Figure 5).

Using ultra deep sequencing analysis, we also dissected the clonal architecture of *MLL-AF4*+ B-cell leukaemia. Among RAS^mut^ MLL-AF4 rearranged leukaemia specimens at diagnosis, 69.5% were characterized by ≥2 different clones with a distinct RAS mutation in the mutation hot spot regions, which also means that *KRAS* and *NRAS* mutations are not mutually exclusive. These results further support previous data on the MLL-AF4 clonality based on the analysis of Ig/TCR rearrangements^40^, which overall depicts MLL-AF4+ leukaemia as a highly heterogeneous and subclonal disease.

Moreover, in line with previous data^23^, a higher frequency of *RAS* mutation was detected in infants rather than in older patients, corroborating the concept that aberrant RAS expression might shorten leukaemia latency^11^.

Nevertheless, all mutations detected at diagnosis had a VAF well below 50%, suggesting that only minor clones carry *RAS* genetic alterations and, in agreement with recent reports^13^,^23^,^37^,^41^, pointing to *RAS* mutations as secondary genetic events. However, the subclonal nature of *KRAS* and *NRAS* activating mutations is not just an unusual feature in cancer; low *RAS* mutation load was reported in colorectal cancer biopsies^42^, early T-cell precursor acute lymphoblastic leukaemia (ETP-ALL)^43^ and also in acute myeloid leukaemia (AML) with *MLL* rearrangements^44^.

Intriguing results were also obtained analysing the RAS^mut^ clones during the disease progression. The analysis of seventeen matched diagnosis-relapse samples revealed that 50% of the mutated patients at diagnosis carried also *RAS* mutations at relapse, pointing to *RAS* mutations as a recurrent genetic aberration not only at diagnosis but also at relapse. Even more interestingly, 6 out of 7 RAS^mut^ patients at relapse carried the same RAS mutated clone detected at diagnosis, which suggests that at least one clone resisted chemotherapy and survived the therapy-related selection pressure (Fig. 2B, cluster 2), although a fractions of mutated clones at diagnosis were targeted by the chemotherapy treatment and disappeared at relapse. De *novo* mutated clones (n = 4) (Fig. 2C, cluster 3) were also identified at relapse in 2 patients, further supporting the notion of *RAS* mutations as secondary events. Moreover, even though only 50% of the analysed paired samples presented a mutation also at relapse, a percentage that still raises some questions about the overall role of *RAS* mutations in MLL-AF4+ leukaemia, the mutated cells with a total VAF >10% seemed to show a certain dependency on the presence of an active *RAS* signalling, which may suggest that *RAS* mutations can sustain proliferative pathways already active in the leukaemic cells. MLL-AF4 leukaemia is known to be characterized by high *FLT3* expression^16^ which results in the activation of its downstream proliferative pathways such as RAS/MAP2K1/MAPK1 and the PIK3CA/AKT1 pathways^45^. We did not identify a significant correlation between *FLT3* expression and *RAS* VAF in RAS^mut^ patients (Supplementary Figure 6); however, we could not exclude the hypothesis that *RAS* mutations may still complement existing proliferative signalling pathways, thus providing support to cells’ survival.

Moreover, our analysis of RAS^mut^ patients at diagnosis highlighted the over-expression of RAS down-stream targets^46^ proving, in line with previous observations from transcriptome analysis^13^, the functionality of RAS signalling in these specimens, which is totally independent from the HOXA genes’ signature^24^.

Overall, the high percentage of RAS^mut^ patients at diagnosis, the presence of an active RAS-related signature at diagnosis as well as the detection of therapy resistant RAS mutated clones in 6/7 (85.7%) mutated samples at relapse indicated that RAS-pathway inhibition might represent a promising approach for a MLL-AF4+ RAS mutated patient-tailored treatment. Direct targeting of RAS has been evaluated in diseases coined “RASopathies” all associated with RAS mutations but without any successes so far^47^. Conversely, preclinical studies evaluating the inhibition of RAS-downstream molecules such as RAS/MAP2K1/2 and MAPK3/1 or PIK3CA/AKT1 have provided promising results^47^,^48^. Here, we successfully established for the first time, to our knowledge, a RAS^mut^ BCP-MLL-AF4 positive leukaemia cell line and we showed that the allosteric MAP2K inhibitor PD0325901^49^ at nanomolar concentration reduced the RAS^mut^ MLL-AF4+ B cell line proliferation to 40% without any significant effect on RAS^wt^ MLL-AF4+ B cell lines even at micromolar concentration, suggesting to evaluate RAS-pathway inhibition by PD0325901 in MLL-AF4+ RAS^mut^ specimens in preclinical studies. These results are in line with published data on *RAS* mutant paediatric B-ALL samples (not *MLL* rearranged) that were treated with two different MAP2K1/MAPK1 inhibitors (i.e. PD98059 and U0126) showing higher cytotoxicity in the mutant samples and no effects in the RAS^wt^ specimens^50^.

In conclusion, taking advantage of next generation sequencing technology we demonstrated that *RAS* mutations are very frequent in MLL-AF4+ leukaemia especially in infant patients. Moreover, the analyses on paired diagnosis-relapse samples indicate that even if current therapy regimens can eliminate some of the *RAS* mutated clones, they do not remove all clones but rather allow the positive selection of the most resistant ones at relapse. Therefore, the potential use of *RAS* pathway inhibitors such as PD0325901 could be considered for precision of current therapy.

## Materials and Methods

### Patient samples, cell lines and xenotransplantation

DNA was isolated from bone marrow (BM) and/or peripheral blood (PB) of 36 paediatric patients diagnosed with MLL-AF4 positive B cell precursor (BCP) ALL and participating to the Interfant-99 (n = 10), Interfant-06 (n = 13) and AIEOP-BFM ALL-2000 (n = 13) treatment protocols. Patients’ characteristics are reported in Table 1. The present study has been carried out in accordance with ethical principles of the Declaration of Helsinki. The residual patients’ material (such as mononuclear cells from PB and BM, DNA and RNA) retained after the completion of the diagnostic screenings has been used for research purposes. The written informed consent was obtained from the patients’ parents or legal guardians. The use of primary patient samples was approved by the local Ethics Committee and by the scientific board of the Interfant Protocol (www.oncauvergne.fr/index.php?option=com_docman&task=doc_download&gid=944&Itemid). The methods were carried out in accordance with the approved guidelines and the samples used for analysis were selected according to material availability.

The MLL-AF4 positive cell lines RS4;11 and SEM were purchased from the German Collection of Microorganisms and Cell Cultures (DSMZ, Braunschweig, Germany) and were used as RAS wild type (RAS^wt^) controls. The MLL-AF9 positive myeloid cell line THP-1 was also purchased from DSMZ and was used as a positive RAS^mut^ control. Information regarding the RAS mutational status of RS4;11, SEM and THP-1 cell lines was collected from the Cancer Cell Line Encyclopedia (CCLE) (http://www.broadinstitute.org/ccle/home). A RAS mutated (RAS^mut^) BCP-ALL *MLL-AF4* positive cell line (named MI04) was successfully established culturing the BM mononuclear cells of a patient diagnosed with MLL-AF4+ BCP ALL. Outgrowing cells were initially cultured in the presence of autologous BM stromal cells. After few weeks cell line growth became independent from the presence of the stromal cell layer. Cell culture conditions are reported below. The presence of the *MLL-AF4* and *AF4-MLL* translocations and of the *N-RAS* Q61R mutation in the newly established cell line were assessed by FISH using a MLL/AFF1 dual fusion probe (Cytocell, United Kingdom) and Sanger Sequencing, respectively (Supplementary Figure 7). All cell lines were maintained as suspension cultures in RPMI 1640 (Invitrogen Life Technologies, The Netherlands) supplemented with 10% fetal calf serum (FCS), glutamine (2 mM/l; GIBCO, Invitrogen Life Technologies, Carlsbad, CA, USA), penicillin (100 U/ml; GIBCO) and streptomycin (100 μg/ml; GIBCO), and maintained at 37 °C in a humidified atmosphere with 5% CO_2_. Cells were treated with PD0325901 (Selleckchem, Houston, TX, USA) dissolved in dimethylsulphoxide (DMSO) for 72 hours and at different concentrations, or with DMSO alone.

Bone marrow cells from patient 35 at diagnosis were serially transplanted into sublethally irradiated NOD/SCID mice via tail vein injection, as previously described^40^. Upon signs of illness, mice were sacrificed and BM cells collected for DNA extraction. The *in vivo* procedures were performed in the Animal Facility at the University of Milano-Bicocca, under the protocol nr. 64/2014-PR approved by the National Ministry of Public Health in accordance with the relevant guidelines and in compliance with the National law (D. Lgs. n. 26/2014) and the European Directive (2010/63/UE) about the protection of live animals used for scientific purposes.

### Amplicon ultra deep sequencing preparation

DNA was extracted using the Gentra Puregene DNA isolation kit (QIAGEN, Hilden, Germany); 20 ng/ul of genomic DNA were processed for the generation of PCR amplicons for ultra deep sequencing, according to manufacture’ protocol (Roche Applied Science, Penzberg, Germany). Fusion primers with 6 different molecular identifier barcode sequences (MID) were designed to amplify hot spot regions of exon 2 and exon 3 of *KRAS* and *NRAS* genes (Supplementary Table 7), for a total of 72 different amplicons for each gene. PCRs were conducted using FastStart High Fidelity PCR System kit (Roche Applied Science). Amplicon products were purified using Agencourt AMPure XP beads (Beckman Coulter, Krefeld, Germany), quantified using the Quant-iT PicoGreen dsDNA kit (Invitrogen, Carlsbad, CA, USA) and equimolar pooled together for the emPCR-Lib-A method (library at 1 × 10^7^ molecules/ul). For each amplicon forward (A beads) and reverse (B beads) reactions were carried out using 5.000.000 beads per emulsion oil tube. The amplification reaction, breaking of the emulsions and enrichment of beads carrying amplified DNA were performed using the workflow as recommended by the manufacturer (Roche Applied Science).

### Data analysis and variants detections

Data analysis was performed using the Roche proprietary software package for the GS Junior system (http://www.454.com/). Sequencing data was generated using GS Junior Sequencer Instrument software version 2.7 (Roche Applied Science). Image processing was performed using default settings of the GS RunBrowser software version 2.7 (Roche Applied Science). We generated a median of ~75.000 and ~62.000 sequencing reads for *KRAS* and *NRAS* obtaining a median of 1540- and 1762-fold coverage for *KRAS* and *NRAS* amplicons.

Sequence alignment and variant detection were performed using the GS Amplicon Variant Analyzer (AVA) software version 2.7 (Roche Applied Science). This software extracts sequences from the standard flowgram format (SFF) files generated after pyrosequencing and automatically assigns each read to the proper sample by looking for the MIDs located at both ends of amplicon. Only sequences with an average phred equivalent quality score >Q20 were conserved. The MIDs and primer sequences within the read have also to be complete without mismatch. Moreover, the reads have to match the full-length amplicon. AVA application computes the alignment of filtered reads from amplicon libraries to identify differences between the reads and the reference sequences (RefSeq NM_004985, and NM_002524, for *KRAS* and *NRAS*, respectively) to call variants. The Variant/Consensus parameters of AVA software were set to minimum read percentage of 0.01% (per read direction), minimum read count of “1” per orientation and appearing in both forward and reverse directions, and dynamic N-mer thresholding for homopolymers. Variant Allele Frequencies (VAFs) of variant *i* were measured as the number of reads carrying the variation *i*, divided by the number of all reads spanning the position of the variant.

In order to establish a bioinformatic approach to identify the best threshold for *KRAS* and *NRAS* subclonal mutations out of the background error noise of ultra-deep-NGS, a triplicate experiment was performed for a subset of patients. Variants across the entire sequence of amplicons were analysed and evaluated for recurrence in all triplicate samples. Only variants presented in all triplicate were considered as true positive call. Variants were divided in different intervals thresholds (i.e. 0.01–0.1; 0.1–03; 0.3–0.5; 0.5–1; 1–5; >5) to estimate the best cut-off with minor false positive call. Variants overlapping the primers or neighbouring them by one nucleotide and variants mapping within homopolymeric tracts that specifically suffer from errors in 454 NGS^51^,^52^ were excluded from the analysis. The same approach was used considering only variants occurring in hot spot loci. Variants with a VAF of 1% and more in bidirectional reads per amplicon were considered.

Different variants for a specific genomic position in the same amplicon were considered as different clone. The mutated clones’ dynamics between diagnosis and relapse were defined in clusters as follows: Cluster 1: variants detected at diagnosis and disappeared at relapse; Cluster 2: all variants detected both at diagnosis and at relapse with similar or increased VAF; Cluster 3: *de novo* variants not present at diagnosis but appearing at relapse.

### Gene expression analysis

Gene expression profiles (GEPs) of patients included in this study were available as a part of the MILE study^53^. For the gene expression analysis only *MLL-AF4* positive patients with available *RAS* mutational status information and high *HOXA9* and *HOXA10* expression levels were used. Data analysis was performed in R (http://www.R-project.org/ version 3.0.2) using Bioconductor and R packages. Probe level signals were normalized and converted to expression values using the robust multi-array averaging (RMA) algorithm^54^. After normalization, batch effects were removed due to different protocols using ComBat method^55^. Differentially expressed genes (FDR q-val < 0.05) were identified using the Shrinkage t-statistic^56^. Gene set enrichment analysis (GSEA) was done comparing the expression profiles of RAS^mut^ and RAS^wt^ samples using the “C5 molecular function” and “C6 oncogenic signatures” gene sets within the molecular signatures databases (MSigDB) collection^57^. The signal to noise metric and the gene-set permutation were used to identify statistical enrichment of the selected gene sets. Enrichment maps showing relationships between gene sets with significant enrichment (p-value cutoff < 0.005; FDR q-val < 0.05; overlap coefficient: 0.5) were generated using the Enrichment Map plug-in in Cytoscape 3.2.1^58^. Only Gene sets with FDR <0.05 derived from the “C6 oncogenic signatures” and the “C5 molecular function” within the MSigDB were used to build the network. To generate gene-set relationships, we used the overlap coefficient cut-off parameter set to 0.5. A node represents a functional gene set and the size is proportional to size of gene set. Edges represent the degree of gene overlap between two gene sets and the thickness is proportional to the overlap between the gene sets. The intensity of the node colour is proportional to the enrichment score. Connectivity map analysis^59^ was performed using genes differentially expressed (FDR q-val < 0.1) between RAS^mut^ and RAS^wt^ samples.

### Reverse Phase Protein Arrays (RPPA)

Reverse Phase Protein Arrays (RPPA) were performed as previously described^60^. Briefly, for this analysis 10 patients (8 RAS^mut^ and 2 RAS^wt^) were lysed and printed on nitrocellulose coated glass slides using the 2470 Aushon Arrayer (Aushon BioSystems, Billerica, MA, USA). Slides were stained with the following antibodies on an automated slide stainer (Dako Autostainer Plus, Dako Cytomation, Carpinteria, CA, USA) using the CSA kit (Dako Cytomation): AKT1 S473 (1:100), AKT1 T308 (1:100), MTOR S2448 (1:100), RPS6KB1 T389 (1:200), PDPK1 S241 (1:100), EIF4G1 S1108 (1:100), GSK3A/B S21/9 (1:100), MAP2K1/2 S217/221 (1:500), MAPK3/MAPK1 T202/Y204 (1:500), PKCD T505 (1:75) (all from Cell Signaling Technology Inc, Danvers, MA, USA), PKCA S657 (1:2500) (Merck Millipore, Darmstadt, Germany), YAP1 S127 (1:100) and EIF4EBP1 S65 (1:250) (Abcam, Cambridge, UK), and AKT1S1 T246 (1:1000) (Thermo Fischer Scientific, Waltham, MA, USA). Stained slides were analysed using the MicroVigene software (VigeneTech Inc, Boston, MA, USA), normalizing for total protein content quantified by Fast Green FCF (Sigma-Aldrich, St Louis, MO, USA) staining. Normalized protein values were used for an unsupervised hierarchical clustering analysis using the Ward’s method in R (version 2.14.1).

### MTT assay

Cell proliferation was assessed by MTT ((3-(4,5-dimethylthiazol-2-yl)-2,5-diphenyl tetrazolium bromide) assay after treatment. Equal concentrations of cells were plated in quadruplicate in a 96-well plate and incubated with 10 μl MTT (Sigma-Aldrich) for 4h. Absorbance was measured at 560 nm using Victor3TM 1420 Multilabel Counter (PerkinElmer, Waltham, MA, USA).

### Statistical analysis

Event-free survival (EFS) was defined as the time from diagnosis to first event, i.e. resistance, relapse, death from any cause, or second malignant neoplasm. Observation periods were censored at time of last contact when no events were reported. EFS curves were estimated with the Kaplan-Meier method and standard errors according to Greenwood and compared with the log-rank test. Cumulative incidence of relapse (CIR) curves were estimated adjusting for competing risks and compared with Gray’s test. Fisher’s exact test was used to assess the association between clinical characteristics and occurrence of RAS mutations. All tests were two sided. Analyses were performed using SAS 9.2. Correlation analysis and Mann-Whitney test were performed using Prism 5 for MAC Version 5.0a; p- values < 0.05 were considered significant.

## Additional Information

**Accession Codes:** Gene expression data are deposited in the GEO database under accession number GSE77416.

**How to cite this article**: Trentin, L. *et al*. Deciphering *KRAS* and *NRAS* mutated clone dynamics in MLL-AF4 paediatric leukaemia by ultra deep sequencing analysis. *Sci. Rep.* **6**, 34449; doi: 10.1038/srep34449 (2016).

## Supplementary Material

Supplementary Information

## Figures and Tables

**Table 1:** Patients characteristics.

	Infants			Children >1 year			Overall			
	RAS neg	RAS pos	Tot	RAS neg	RAS pos	Tot	RAS neg	RAS pos	Tot	p-value^*^
N. pts.	5	17	22	8	4	12	13	21	34	
Sex										
M	2	*9*	11	3	*1*	4	5	*10*	15	0.7282
F	3	*8*	11	5	*3*	8	8	*11*	19	
Age at diagnosis										
0–5 months	4	*13*	17	—	—	—	4	*13*	17	0.0248^§^
6–12 months	1	*4*	5	—	*—*	—	1	*4*	5	
1–5 years	—	—	—	4	*3*	7	4	*3*	7	
≥6 years	—	—	—	4	*1*	5	4	*1*	5^^^	
WBC counts (cell/L)										
<100 × 10^−9^	0	*1*	1	3	*1*	4	3	*2*	5	0.4720
100–300 × 10^−9^	2	*5*	7	2	*0*	2	4	*5*	9	
≥300 × 10^−9^	3	*11*	14	3	*2*	5	6	*13*	19	
Not known	0	*0*	0	0	*1*	1	0	*1*	1	
Immunophenotype										
Pro-B	5	*16*	21	7	*4*	11	12	*20*	32	—
Pre-B	0	*1*	1	1	*0*	1	1	*1*	2	
PDN response										
PPR	1	*6*	7	1	*1*	2	2	*7*	9	0.2491
PGR	4	*10*	14	7	*2*	9	11	*12*	23	
Not known	0	*1*	1	0	*1*	1	0	*2*	2	

^^^N = 2 were >10 years at diagnosis (none RAS positive).

*p-values compare RAS positive vs negative overall by relevant characteristics at diagnosis/response (patients with unavailable data on WBC and PDN response are excluded from the respective comparison).

^§^comparison of infants vs >1 year.
