# Supplementary material for: Deciphering KRAS and NRAS mutated clone dynamics in MLL-AF4 paediatric leukaemia by ultra deep sequencing analysis
Source: Sci Rep. 2016 Oct 4;6:34449. doi: 10.1038/srep34449 (PMC5048141; doi:10.1038/srep34449)

Deciphering *KRAS* and *NRAS* mutated clone dynamics in MLL-AF4 paediatric leukaemia by ultra deep sequencing analysis

Luca Trentin[1*], Silvia Bresolin[1], Emanuela Giarin[1], Michela Bardini[2], Valentina Serafin[1], Benedetta Accordi[1], Franco Fais[3,4], Claudya Tenca[3], Paola De Lorenzo[2,5], Maria Grazia Valsecchi[5], Giovanni Cazzaniga[2], Geertruy te Kronnie[1,*] & Giuseppe Basso[1,*]

Supplementary Information

Supplementary Table 1

The table summarizes the *KRAS* and *NRAS* mutation status in MLL-AF4+ patients at diagnosis and at relapse.

Sample	Mutations in *KRAS*	Mutations in *NRAS*	Total Clones (D)	VAF Index [%] (D)	GEP Data (D)	RPPA Data (D)	Paired D-R	Clonal evolution			Total Clones (R)	VAF Index [%] (R)
								Cluster1	Cluster 2	Cluster 3		
Pt 1	G12S	G12D*<br>Q61P	3	31.75	no	yes	no^	-	-	-	-	-
Pt 2		Q61L	1	23.83	no	no	no^	-	-	-	-	-
Pt 3			0	0	no	no	no^	-	-	-	-	-
Pt 4	G13D		1	19.18	no	no	yes	G13D	-	-	0	0
Pt 5	G12S*<br>G13D		2	15.15	yes	no	no^^	-	-	-	-	-
Pt 6				0	no	yes	no^^	-	-	-	-	-
Pt 7				0	no	no	no^^	-	-	-	-	-
Pt 8				0	yes	no	no^^	-	-	-	-	-
Pt 9	G12V*	G12D*<br>G13D*		5.47	no	yes	yes	G12V<br>G12D<br>G13D	-	-	0	0
Pt 10		G12D*<br>G13D	3	18.92			yes	G12D	G13D	-	1	-
Pt 11	G12S<br>A18D*		2	26.14	no	yes	no^^	-	-	-	-	-
Pt 12		G12D*	1	4.69	yes	no	yes	G12D	-	-	0	0

Sample	Mutations in *KRAS*	Mutations in *NRAS*	Total Clones (D)	VAF Index [%] (D)	GEP Data (D)	RPPA Data (D)	Paired D-R	Clonal evolution			Total Clones (R)	VAF Index [%] (R)
								Cluster1	Cluster 2	Cluster 3		
Pt 13	G12S*<br>G12D*<br>G13D*	G12D*<br>G12A*<br>G13D*	6	39.34	yes	no	no^^	-	-	-	-	-
Pt 14	G12S*<br>G12D	G12S*<br>Q61K*<br>Q61H*	5	37.6	no	yes	no^^	-	-	-	-	-
Pt 15			0	0	yes	no	no^^	-	-	-	-	-
Pt 16			0	0	no	no	yes	-	0	0	0	-
Pt 17		G12S*<br>G12D*	2	4.85	no	no	no^^	-	-	-	-	-
Pt 18	G12D*		1	4.93	yes	no	yes	-	G12D*	G13D*^{KRAS}<br>G12D*^{NRAS}<br>G13D ^{NRAS}	4	20.95
Pt 19	G12R<br>G13D*		2	33.3	yes	yes	no^^	-	-	-	-	-
Pt 20			0	0	yes	no	no^^	-	-	-	-	-
Pt 21	G13D<br>Q61H		2	27.08	yes	no	yes	G13D<br>Q61H	-	-	0	0
Pt 22	G12V*<br>G12D		2	37.84	yes	no	yes	G12V<br>G12D	-	Q61H^{KRAS}	1	37.9
Pt 23			0	0	no	no	no^^	-	-	-	-	-
Pt 24			0	0	yes	no	no^	-	-	-	-	-
Pt 25	G12D*<br>G13D*	Q61K*	3	16.56	yes	no	yes	G12D<br>G13D	Q61K	-	1	84.29
Pt 26	G12D*<br>G13D*		2	6.11	no	yes	yes	G12D<br>G13D	-	-	0	0
Pt 27	G12D*<br>G13D*	G12V*	3	10.18	no	yes	yes	G13D<br>G12V	G12D	-	1	26.2

Sample	Mutations in *KRAS*	Mutations in *NRAS*	Total Clones (D)	VAF Index [%] (D)	GEP Data (D)	RPPA Data (D)	Paired D-R	Clonal evolution			Total Clones (R)	VAF Index [%] (R)
								Cluster1	Cluster 2	Cluster 3		
Pt 28	G12V<br>G12D*	G12D*<br>G13D*	4	28.45	no	no	yes	G12D<br>G12D<br>G13D	G12V	-	1	53.36
Pt 29	G12D		1	17.14	no	no	yes	G12D	-	-	0	0
Pt 30			0	0	no	no	yes	-	0	0	0	0
Pt 31			0	0	no	yes	no^^	-	-	-	-	-
Pt 32			0	0	no	no	no^^	-	-	-	-	-
Pt 33			0	0	no	no	yes	-	0	0	0	0
Pt 34	G12D		1	40	yes	no	yes	-	G12D	-	1	32.59
Pt 35	G12S*		1	2.83	no	yes	yes	G12S	-	-	0	0
Pt 38	G12D*	G13D	2	42.33	yes	no	no^^	-	-	-	-	-

D: diagnosis; R: relapse; VAF Index: total variant allele frequency for all mutated clones at the indicated time point; cluster 1: mutations lost at relapse and present only at diagnosis; cluster 2: mutations with constant/increasing VAF from diagnosis to relapse; cluster 3: mutations acquired *de novo* at relapse; GEP: gene expression profile; RPPA: reverse phase protein array; ^: patient relapsed but no material available; ^^: not relapsed patient; *: mutations with a VAF < 10%.

Supplementary Table 2

Variant allele frequency (VAF) in *KRAS* and *NRAS* exons 2 and 3 at diagnosis.

# Pt	G12S	G12V	G12D	G12R	G13D	A18D	Q61R	Q61H	G12S	G12V	G12D	G12A	G13D	Q61P	Q61L	Q61K	Q61H
1	20.64	0	0	0	0	0	0	0	0	0	1.94	0	0	9.17	0	0	0
2	0	0	0	0	0	0	0	0	0	0	0	0	0	0	23.83	0	0
3	0	0	0	0	0	0	0	0	0	0	0	0	0	0	0	0	0
4	0	0	0	0	19.18	0	0	0	0	0	0	0	0	0	0	0	0
5	2.59	0	0	0	12.56	0	0	0	0	0	0	0	0	0	0	0	0
6	0	0	0	0	0	0	0	0	0	0	0	0	0	0	0	0	0
7	0	0	0	0	0	0	0	0	0	0	0	0	0	0	0	0	0
8	0	0	0	0	0	0	0	0	0	0	0	0	0	0	0	0	0
9	0	2.14	0	0	0	0	0	0	0	0	1.62	0	1.71	0	0	0	0
10	0	0	0	0	0	0	0	0	0	0	1.09	0	17.83	0	0	0	0
11	22.18	0	0	0	0	2.96	1	0	0	0	0	0	0	0	0	0	0
12	0	0	0	0	0	0	0	0	0	0	4.69	0	0	0	0	0	0
13	3.97	0	3.42	0	8.63	0	0	0	0	0	4.75	9.14	9.43	0	0	0	0
14	1.1	0	29.7	0	0	0	0	0	3.61	0	0	0	0	0	0	2.17	1.02

# Pt	G12S	G12V	G12D	G12R	G13D	A18D	Q61R	Q61H	G12S	G12V	G12D	G12A	G13D	Q61P	Q61L	Q61K	Q61H
15	0	0	0	0	0	0	0	0	0	0	0	0	0	0	0	0	0
16	0	0	0	0	0	0	0	0	0	0	0	0	0	0	0	0	0
17	0	0	0	0	0	0	0	0	2.8	0	2.05	0	0	0	0	0	0
18	0	0	4.93	0	0	0	0	0	0	0	0	0	0	0	0	0	0
19	0	0	0	27.11	6.19	0	0	0	0	0	0	0	0	0	0	0	0
20	0	0	0	0	0	0	0	0	0	0	0	0	0	0	0	0	0
21	0	0	0	0	10.06	0	0	17.02	0	0	0	0	0	0	0	0	0
22	0	7.14	30.7	0	0	0	0	0	0	0	0	0	0	0	0	0	0
23	0	0	0	0	0	0	0	0	0	0	0	0	0	0	0	0	0
24	0	0	0	0	0	0	0	0	0	0	0	0	0	0	0	0	0
25	0	0	6.49	0	1.72	0	0	0	0	0	0	0	0	0	0	8.35	0
26	0	0	5.06	0	1.05	0	0	0	0	0	0	0	0	0	0	0	0
27	0	0	3.47	0	2.61	0	0	0	0	4.1	0	0	0	0	0	0	0
28	0	13.89	3.24	0	0	0	0	0	0	0	9.67	0	1.65	0	0	0	0
29	0	0	17.14	0	0	0	0	0	0	0	0	0	0	0	0	0	0
30	0	0	0	0	0	0	0	0	0	0	0	0	0	0	0	0	0
31	0	0	0	0	0	0	0	0	0	0	0	0	0	0	0	0	0

# Pt	G12S	G12V	G12D	G12R	G13D	A18D	Q61R	Q61H	G12S	G12V	G12D	G12A	G13D	Q61P	Q61L	Q61K	Q61H
32	0	0	0	0	0	0	0	0	0	0	0	0	0	0	0	0	0
33	0	0	0	0	0	0	0	0	0	0	0	0	0	0	0	0	0
34	0	0	40	0	0	0	0	0	0	0	0	0	0	0	0	0	0
35	2.83	0	0	0	0	0	0	0	0	0	0	0	0	0	0	0	0
38	0	0	3.85	0	0	0	0	0	0	0	0	0	38.48	0	0	0	0

Dark gray: *KRAS*; light gray: *NRAS*

Supplementary Table 3 Patients' outcome.

	Infants			Children >1 year			Overall		
	**RAS neg**	**RAS pos**	**Tot**	**RAS neg**	**RAS pos**	**Tot**	**RAS neg**	**RAS pos**	**Tot**
**N. pts.**	**5**	**17**	**22**	**8**	**4**	**12**	**13**	**21**	**34**
**N. events**	3	*15*	18	5	*3*	8	8	*18*	26
**Resistant to Induction**	0	*0*	0	0	*1*	1	0	*1*	1
**Relapse**	2	*13*	15	4	*2*	6	6	*15*	21
BM	2	*12*	14	2	*2*	4	4	*14*	18
Testis	0	*0*	0	1	*0*	1	1	*0*	1
BM+CNS	0	*1*	1	0	*0*	0	0	*1*	1
Mediastinal	0	*0*	0	1	*0*	1	1	*0*	1
**Death in CCR**									
SCT-related	1	*2*	3	1	*0*	1	2	*2*	4
Not known	0	*0*	0	1	*0*	1	1	*0*	1

Supplementary Table 4

Variant allele frequency (VAF) in *KRAS* and *NRAS* exons 2 and 3 in paired diagnosis and relapse samples.

# Pt	G12S	G12V	G12D	G12R	G13D	A18D	Q61R	Q61H	G12S	G12V	G12D	G12A	G13D	Q61P	Q61L	Q61K	Q61H
4 D	0	0	0	0	19.18	0	0	0	0	0	0	0	0	0	0	0	0
4 R	0	0	0	0	0	0	0	0	0	0	0	0	0	0	0	0	0
9 D	0	2.14	0	0	0	0	0	0	0	0	1.62	0	1.71	0	0	0	0
9 R	0	0	0	0	0	0	0	0	0	0	0	0	0	0	0	0	0
10 D	0	0	0	0	0	0	0	0	0	0	1.09	0	17.83	0	0	0	0
10 R	0	0	0	0	0	0	0	0	0	0	0	0	31.55	0	0	0	0
12 D	0	0	0	0	0	0	0	0	0	0	4.69	0	0	0	0	0	0
12 R	0	0	0	0	0	0	0	0	0	0	0	0	0	0	0	0	0
16 D	0	0	0	0	0	0	0	0	0	0	0	0	0	0	0	0	0
16 R	0	0	0	0	0	0	0	0	0	0	0	0	0	0	0	0	0
18 D	0	0	4.93	0	0	0	0	0	0	0	0	0	0	0	0	0	0
18 R	0	0	1.31	0	3.3	0	0	0	0	0	1.04	0	15.3	0	0	0	0
21 D	0	0	0	0	10.06	0	0	17.02	0	0	0	0	0	0	0	0	0
21 R	0	0	0	0	0	0	0	0	0	0	0	0	0	0	0	0	0
22 D	0	7.14	30.7	0	0	0	0	0	0	0	0	0	0	0	0	0	0
22 R	0	0	0	0	0	0	0	37.9	0	0	0	0	0	0	0	0	0
25 D	0	0	6.49	0	1.72	0	0	0	0	0	0	0	0	0	0	8.35	0
25 R	0	0	0	0	0	0	0	0	0	0	0	0	0	0	0	84.29	0

# Pt	G12S	G12V	G12D	G12R	G13D	A18D	Q61R	Q61H	G12S	G12V	G12D	G12A	G13D	Q61P	Q61L	Q61K	Q61H
26 D	0	0	5.06	0	1.05	0	0	0	0	0	0	0	0	0	0	0	0
26 R	0	0	0	0	0	0	0	0	0	0	0	0	0	0	0	0	0
27 D	0	0	3.47	0	2.61	0	0	0	0	4.1	0	0	0	0	0	0	0
27 R	0	0	26.2	0	0	0	0	0	0	0	0	0	0	0	0	0	0
28 D	0	13.89	3.24	0	0	0	0	0	0	0	9.67	0	1.65	0	0	0	0
28 R	0	53.36	0	0	0	0	0	0	0	0	0	0	0	0	0	0	0
29 D	0	0	17.14	0	0	0	0	0	0	0	0	0	0	0	0	0	0
29 R	0	0	0	0	0	0	0	0	0	0	0	0	0	0	0	0	0
30 D	0	0	0	0	0	0	0	0	0	0	0	0	0	0	0	0	0
30 R	0	0	0	0	0	0	0	0	0	0	0	0	0	0	0	0	0
33 D	0	0	0	0	0	0	0	0	0	0	0	0	0	0	0	0	0
33 R	0	0	0	0	0	0	0	0	0	0	0	0	0	0	0	0	0
34 D	0	0	40	0	0	0	0	0	0	0	0	0	0	0	0	0	0
34 R	0	0	32.59	0	0	0	0	0	0	0	0	0	0	0	0	0	0
35 D	2.83	0	0	0	0	0	0	0	0	0	0	0	0	0	0	0	0
35 R	0	0	0	0	0	0	0	0	0	0	0	0	0	0	0	0	0

Dark gray: *KRAS*; light gray: *NRAS*; patient at diagnosis (D): blank background; patient at relapse (R): dotted background.

Supplementary Table 5

Differentially expressed genes between RAS^{wt} and RAS^{mut} samples identified according to Shrinkage t test statistic using a FDR q-val < 0.05. FC: fold change. The FC is reported as the ratio between the two considered group means.

probe set	gene symbol	q-value	means_RAS^{wt}	means_RAS^{mut}	FC
217820_s_at	*ENAH*	4.55E-10	2.922552656	5.870065214	0.497874
234986_at	*GCLM*	0.041366	2.915739143	4.97053853	0.586604
222433_at	*ENAH*	2.39E-11	3.687893998	6.034829169	0.611102
212458_at	*SPRED2*	0.033408	2.95012527	4.816165271	0.612547
204529_s_at	*TOX*	0.000256	4.587803501	7.1870007	0.638347
238756_at	*GAS2L3*	7.63E-05	4.345925101	6.768292878	0.642101
217853_at	*TNS3*	0.00473	5.246660315	8.131337038	0.64524
226837_at	*SPRED1*	0.00561	2.94090845	4.544142345	0.647187
212158_at	*SDC2*	0.032592	5.697409978	8.285445336	0.687641
212985_at	*APBB2*	0.019043	4.090535301	5.912763968	0.691814
213848_at	*DUSP7*	0.002077	4.034616918	5.782011702	0.697788
218035_s_at	*RBM47*	7.19E-07	4.828983374	6.587712024	0.733029
202599_s_at	*NRIP1*	0.000443	7.026982623	9.547539781	0.735999
231835_at	*FAM213B*	0.00078	4.403641404	5.971492205	0.737444
204192_at	*CD37*	0.008505	6.016083852	7.94630087	0.757092
235072_s_at	*KIF13A*	0.011471	3.700820167	4.820138393	0.767783
221658_s_at	*IL21R*	0.014294	4.975847401	6.477477121	0.768177
1555613_a_at	*ZAP70*	0.009382	4.559580374	5.929756832	0.768932
202600_s_at	*NRIP1*	0.000412	7.480677021	9.694800047	0.771617
222496_s_at	*RBM47*	0.036772	5.061524406	6.539705239	0.773968
219686_at	*STK32B*	0.003293	4.342432546	5.601064606	0.775287
213135_at	*TIAM1*	0.014867	4.100756455	5.197934504	0.78892
225869_s_at	*UNC93B1*	4.06E-05	4.769127039	5.955185358	0.800836
237753_at	*IL21R*	0.000199	4.287917222	5.30674827	0.808012
225214_at	*LOC100129034*	0.000353	5.872166165	7.257598011	0.809106
217422_s_at	*CD22*	0.049178	3.762721032	4.639171154	0.811076
204681_s_at	*RAPGEF5*	0.046123	4.83141112	5.947144607	0.812392
228442_at	*NFATC2*	0.001846	4.798644746	5.895868334	0.8139
212157_at	*SDC2*	0.008407	4.40457585	5.395495024	0.816343
226765_at	*SPTBN1*	0.018766	4.785671053	5.857764558	0.816979
229249_at	*NA*	0.032649	4.287584477	5.247251957	0.81711
229250_at	*TPCN2*	0.000439	4.508245833	5.463666516	0.825132
216037_x_at	*TCF7L2*	0.032672	5.666220358	6.85368506	0.826741
236295_s_at	*NLRC3*	0.025289	4.601071206	5.534949496	0.831276
209321_s_at	*ADCY3*	0.006675	4.72425843	5.680425114	0.831673
228955_at	*LRP8*	0.024742	5.728527101	6.887694125	0.831705
1557919_a_at	*ERVH-3*	0.026954	4.074221455	4.874529853	0.835818

221643_s_at	*RERE*	0.024891	3.196368156	3.823455108	0.835989
227401_at	*IL17D*	0.007779	4.070454801	4.855266478	0.838359
222116_s_at	*TBC1D16*	0.020188	5.640526228	6.72401448	0.838863
208820_at	*PTK2*	0.015449	5.525595584	6.58257248	0.839428
204620_s_at	*VCAN*	0.007772	6.117724775	7.271159108	0.841369
244261_at	*IFNLR1*	0.001985	6.603064878	7.842534757	0.841955
218947_s_at	*MTPAP*	0.000584	5.618154003	6.656619747	0.843995
238519_at	*NA*	0.024033	5.705448932	6.759033735	0.844122
203607_at	*INPP5F*	0.024934	5.116893768	6.05879873	0.844539
224925_at	*PREX1*	0.0041	7.087578911	8.369824425	0.846801
235709_at	*GAS2L3*	0.045152	5.28856566	6.224716998	0.849607
204430_s_at	*SLC2A5*	0.015665	6.078978445	7.126438661	0.853018
224393_s_at	*CECR6*	0.041689	4.757979731	5.565894894	0.854845
226983_at	*ZNF777*	0.02257	3.08512392	3.598998994	0.857217
212512_s_at	*NA*	0.028578	6.315331218	7.347297637	0.859545
216232_s_at	*GCN1L1*	0.045763	5.366187364	6.226529372	0.861826
1558517_s_at	*LRRC8C*	0.00143	4.942829694	5.723384911	0.86362
242866_x_at	*POU2F2*	0.024056	5.086724748	5.869974264	0.866567
209624_s_at	*MCCC2*	0.037925	4.180361154	4.801507064	0.870635
215235_at	*SPTAN1*	0.008684	7.3239968	8.404331088	0.871455
220280_s_at	*ANKMY1*	0.018342	3.975851252	4.552141832	0.873402
221881_s_at	*CLIC4*	0.03154	5.489165426	6.274079888	0.874896
224909_s_at	*PREX1*	0.030796	8.1355779	9.253420311	0.879197
221853_s_at	*NA*	0.035667	6.755198091	7.664228575	0.881393
227677_at	*JAK3*	0.036834	6.859764429	7.781823402	0.881511
222409_at	*CORO1C*	0.02035	7.836001618	8.88539733	0.881897
221861_at	*ARRB1*	0.004776	5.44800844	6.142934947	0.886874
204153_s_at	*MFNG*	0.047006	7.558241712	8.520207188	0.887096
212261_at	*GIGYF2*	0.035178	5.74728992	6.463600812	0.889178
225775_at	*TSPAN33*	0.000736	7.141456984	8.00832587	0.891754
226991_at	*NFATC2*	0.032205	6.205957725	6.957442811	0.891988
203233_at	*IL4R*	0.028702	6.651244065	7.453195531	0.892402
221601_s_at	*FAIM3*	0.020343	6.858928499	7.675474667	0.893616
232149_s_at	*NSMAF*	0.013263	6.274879868	6.994439079	0.897124
225913_at	*PEAK1*	0.001864	5.927752963	6.577964652	0.901153
225929_s_at	*RNF213*	0.025571	6.462403848	7.164875078	0.901956
201767_s_at	*ELAC2*	0.015088	7.002897289	7.746498948	0.904008
200755_s_at	*CALU*	0.043756	6.198959894	6.851695998	0.904734
204245_s_at	*RPP14*	0.045972	4.950198313	5.467683223	0.905356
202477_s_at	*TUBGCP2*	0.037724	6.75752973	7.459116107	0.905942
43511_s_at	*ARRB1*	0.001519	5.814076086	6.409074148	0.907163
212127_at	*RANGAP1*	0.046669	7.031090112	7.745736112	0.907737
200696_s_at	*GSN*	0.029989	9.226943765	10.16428702	0.907781
49111_at	*ARRB1*	0.012738	5.819359818	6.403988326	0.908709
203047_at	*STK10*	0.022939	6.692945525	7.3300858	0.913079
225235_at	*TSPAN17*	0.014501	6.126283151	6.700051991	0.914364

217225_x_at	*NA*	0.04222	8.771766074	9.589416789	0.914734
213160_at	*DOCK2*	0.025826	8.147249588	8.854109981	0.920166
202392_s_at	*NA*	0.04134	6.3275599	6.85905564	0.922512
225398_at	*RPUSD4*	0.039492	7.560820416	8.118522073	0.931305
201037_at	*PFKP*	0.022969	8.518741107	9.10132088	0.93599
212268_at	*SERPINB1*	0.044669	10.43281089	9.932941016	1.050324
1558111_at	*MBNL1*	0.007091	11.12663197	10.36986673	1.072977
238156_at	*NA*	0.04347	8.675038182	8.050665563	1.077555
1557117_at	*INTS6-AS1*	0.045984	5.661227024	5.193501387	1.09006
226413_at	*LINC00938*	0.033705	9.362952715	8.588013159	1.090235
217144_at	*NA*	0.039623	8.556532818	7.838790712	1.091563
1559116_s_at	*NA*	0.046746	4.55877234	4.153217109	1.097648
209492_x_at	*ATP5I*	0.033583	10.38235711	9.438307393	1.100023
226278_at	*SVIP*	0.001611	7.210486552	6.537835358	1.102886
207335_x_at	*ATP5I*	0.033782	9.785672243	8.8563551	1.104932
1558002_at	*STRAP*	0.02816	7.696006265	6.909341938	1.113855
227622_at	*PCF11*	0.005795	8.705463021	7.785813646	1.118119
230847_at	*WRNIP1*	0.005317	7.869646788	7.002075615	1.123902
1559509_at	*NA*	0.045882	3.963360007	3.518583366	1.126408
232716_at	*NA*	0.034966	3.421769829	3.037227753	1.12661
239152_at	*NA*	0.046481	4.921018809	4.358826462	1.128978
225884_s_at	*GZF1*	0.034588	7.765013522	6.873887733	1.129639
239113_at	*LOC100507468*	0.04347	3.508440208	3.103548516	1.130461
244523_at	*MMD*	0.018262	5.938781246	5.245195335	1.132233
242784_at	*NA*	3.96E-08	8.241892141	7.258926663	1.135415
1559132_at	*TMEM80*	0.028952	5.579942762	4.911929813	1.135998
205475_at	*SCRG1*	0.047155	3.832902325	3.367728266	1.138127
203657_s_at	*CTSF*	0.001084	4.900429795	4.28874979	1.142624
224559_at	*MALAT1*	0.032958	8.397598948	7.349153258	1.142662
239213_at	*SERPINB1*	0.024642	6.991387583	6.115862458	1.143156
1568815_a_at	*DDX50*	0.007134	7.459953576	6.514879929	1.145064
1554671_a_at	*SRRM2*	0.000782	7.335057714	6.379984408	1.149698
1566079_at	*NA*	0.039778	8.401792878	7.289684344	1.152559
207184_at	*SLC6A13*	0.017044	4.744432074	4.112538306	1.153651
236851_x_at	*NA*	0.0449	4.864792074	4.210877059	1.155292
228999_at	*CHD2*	7.65E-06	10.30807553	8.898847259	1.158361
236921_at	*NA*	0.034871	9.411578764	8.09229687	1.163029
244443_at	*LINC01578*	0.000477	7.688602696	6.563711886	1.17138
244719_at	*NA*	0.002574	3.939534935	3.356884603	1.173569
1559989_at	*TPM4*	1.44E-06	6.206655293	5.284216619	1.174565
1552665_at	*JMJD1C-AS1*	0.017171	7.778974469	6.621371392	1.174828
238716_at	*LOC100506990*	0.034575	5.545328386	4.71683988	1.175645
241751_at	*OFD1*	7.27E-05	7.50952695	6.35671769	1.181353
243796_at	*NA*	0.041738	6.348631215	5.373489099	1.181473
211360_s_at	*ITPR2*	0.035162	6.15182884	5.195573901	1.184052
1556597_a_at	*LOC284513*	0.047695	4.806117221	4.049861127	1.186736

237464_at	*NA*	5.57E-05	8.506838125	7.150178356	1.189738
236899_at	*NA*	0.03426	5.260940223	4.414102649	1.191848
244035_at	*NA*	0.011737	8.170810213	6.84764374	1.193229
235217_at	*LINC01004*	0.032951	5.588502569	4.67110888	1.196397
209739_s_at	*PNPLA4*	0.010109	5.494966414	4.578287847	1.200223
1558914_at	*DESI2*	0.039963	7.804944361	6.495732272	1.20155
243201_at	*RPL36A*	0.035276	7.435275994	6.174189601	1.204251
236728_at	*LNPEP*	5.68E-05	6.813550909	5.63317943	1.209539
241331_at	*SKAP2*	0.020824	5.117312725	4.22948548	1.209914
235765_at	*TLE4*	0.045037	7.005324536	5.786968828	1.210534
226446_at	*HES6*	0.015321	6.766277484	5.587836373	1.210894
244074_at	*NA*	0.00388	6.099626775	4.991500146	1.222003
210239_at	*IRX5*	0.029474	5.102728804	4.150918726	1.229301
215152_at	*MYB*	0.008701	6.383046944	5.190323904	1.229797
224503_s_at	*ZCCHC2*	0.000415	5.135062329	4.174918484	1.229979
205511_at	*FLJ10038*	0.000631	7.795350053	6.337138181	1.230106
235779_at	*ZNF790-AS1*	0.03089	4.845397353	3.937749246	1.230499
1569076_a_at	*ZNF836*	0.043296	6.047029878	4.91004689	1.231563
1568627_at	*SMEK2*	0.012235	7.794582186	6.291502972	1.238906
1560995_s_at	*NA*	0.020046	5.030916749	4.058413202	1.239627
230670_at	*IGSF10*	0.047986	4.802259779	3.858978139	1.244438
233031_at	*ZEB2*	0.031602	7.489063881	6.003216599	1.247509
236213_at	*NA*	0.040711	6.92903683	5.552920422	1.247818
243683_at	*MORF4L2*	0.033087	6.705220807	5.358760179	1.251263
240256_at	*NA*	0.019662	6.261639105	5.000243148	1.252267
244517_x_at	*NA*	0.002101	5.183546085	4.122909631	1.257254
230636_s_at	*KLF9*	0.000352	4.758991088	3.779324422	1.259217
1560662_s_at	*NA*	0.015376	5.041237512	3.993956977	1.262216
243888_at	*NA*	0.013293	8.459833661	6.688841551	1.264768
237485_at	*SRSF3*	6.27E-06	6.668907534	5.267716788	1.265996
1559964_at	*LOC401261*	0.017161	6.207620619	4.902108429	1.266316
201631_s_at	*IER3*	0.035936	8.01735651	6.317957354	1.268979
243538_at	*NA*	0.035543	5.850469912	4.588038023	1.275157
243206_at	*CEP95*	0.009265	6.895557567	5.39950189	1.277073
1557945_at	*TCTE3*	0.013653	5.077821062	3.973562626	1.277901
226482_s_at	*TSTD1*	0.031967	8.938347555	6.955331265	1.285107
229870_at	*LOC644656*	0.016966	6.867325494	5.305610068	1.294352
242642_at	*NA*	0.009032	3.610109099	2.772216707	1.302246
1552480_s_at	*PTPRC*	0.009103	5.465377307	4.179009457	1.307816
220399_at	*LINC00115*	0.006325	5.575492651	4.247775304	1.312568
229762_at	*NA*	0.005687	6.584103385	5.007256945	1.314912
238501_at	*NA*	0.005859	6.004411496	4.500478043	1.334172
1563113_at	*UBR4*	0.000181	4.986046939	3.732566445	1.335823
39729_at	*PRDX2*	0.039474	8.736439124	6.459231059	1.352551
229795_at	*NA*	2.99E-05	6.191282266	4.553728403	1.359607
1568782_at	*RP2*	0.02977	5.148832014	3.770335353	1.365616

222315_at	*LOC100996756*	0.03216	7.971015384	5.784661008	1.377957
1560486_at	*STXBP3*	0.000338	6.561817768	4.747568019	1.382143
236439_at	*NA*	0.001897	5.37979578	3.884619806	1.384896
231247_s_at	*LOC727820*	0.04119	4.728984286	3.400629785	1.39062
205382_s_at	*CFD*	0.008626	7.98498285	5.737246348	1.39178
236780_at	*NA*	0.002798	5.511429361	3.952335752	1.394474
244546_at	*CYCS*	0.041257	4.981631383	3.553227956	1.402002
1554676_at	*SRGN*	0.008595	6.601321053	4.652961789	1.418735
221766_s_at	*FAM46A*	0.000268	8.293438808	5.831993763	1.422059
1554892_a_at	*MS4A3*	0.007363	7.001963341	4.853799284	1.442574
210254_at	*MS4A3*	0.006883	9.003268645	6.224198806	1.446494
1556545_at	*NA*	0.008268	7.634371786	5.232556249	1.459014
241462_at	*NA*	0.033233	6.076449601	4.062950074	1.495576
232629_at	*PROK2*	7.53E-05	6.652120013	4.394169523	1.513851
224973_at	*FAM46A*	0.001474	4.605793113	2.818279729	1.634257
228170_at	*OLIG1*	0.009098	5.353776886	3.272374741	1.636053
237058_x_at	*SLC6A13*	0.026501	6.44045023	3.821746416	1.685211
239963_at	*NA*	0.009351	7.101981717	3.145326883	2.257947

Supplementary Table 6

Connectivity Map (CMAP) permutated results using the genes differentially expressed between RAS^{mut} and RAS^{wt} patients with a FDR q-val < 0.1. Listed the fifteen molecules best connected (positively and negatively) with the query signature. Negative enrichment scores indicate compounds that can induce a reversal or repression of the biological state encoded in the analyzed gene signature. §: compounds known as related with a RAS mutated phenotype alteration.

rank	cmap name	mean	n	enrichment	p	specificity
1	anisomycin	-0.623	4	-0.964	0	0.0085
2	trichostatin A	0.227	182	0.454	0	0.4123
3	valproic acid	0.236	57	0.333	0	0.0263
4	lycorine §	-0.532	5	-0.895	0.00004	0
5	cephaeline	-0.42	5	-0.838	0.0003	0.0663
6	acebutolol	0.447	5	0.827	0.00032	0
7	cicloheximide §	-0.498	4	-0.886	0.00038	0.0444
8	ouabain §	-0.427	4	-0.866	0.00062	0.0263
9	mometasone	-0.377	4	-0.844	0.00105	0.0086
10	1.4-chrysenequinone	-0.48	2	-0.978	0.00107	0
11	enoxacin §	-0.358	4	-0.819	0.00203	0
12	etiocholanolone	0.29	6	0.687	0.00242	0.0317
13	ofloxacin	-0.335	5	-0.721	0.0036	0.0058
14	glafenine	0.43	4	0.787	0.004	0.0284
15	helveticoside	-0.366	6	-0.662	0.00411	0.026

Supplementary Table 7

KRAS Primers

**Universal adapter_Fw**	CGTATCGCCTCCCTCGCGCCATCAG
**Universal adapter_Rw**	CTATGCGCCTTGCCAGCCCGCTCAG
**MID1**	ACGAGTGCGT
**MID2**	ACGCTCGACA
**MID3**	AGACGCACTC
**MID4**	AGCACTGTAG
**MID5**	ATCAGACACG
**MID6**	ATATCGCGAG
	**Exon 2**
**Fw**	AAAAGGTACTGGTGGAGTATTTGA
**Rw**	CATGAAAATGGTCAGAGAAACC
	**Exon 3**
**Fw**	CCAGACTGTGTTTCTCCCTTC
**Rw**	TGCATGGCATTAGCAAAGAC

NRAS Primers

**Universal adapter_Fw**	CGTATCGCCTCCCTCGCGCCATCAG
**Universal adapter_Rw**	CTATGCGCCTTGCCAGCCCGCTCAG
**MID1**	ACGAGTGCGT
**MID2**	ACGCTCGACA
**MID3**	AGACGCACTC
**MID4**	AGCACTGTAG
**MID5**	ATCAGACACG
**MID6**	ATATCGCGAG
	**Exon 2**
**Fw**	TGGAAGGTCACACTAGGGTT
**Rw**	GGGTAAAGATGATCCGACAAGTG
	**Exon 3**
**Fw**	ATTGAACTTCCCTCCCTCCC
**Rw**	GCTCTATCTTCCCTAGTGTGGT

Supplementary Figure 1

Line graphs showing the percentage of mutations grouped according to six different VAF intervals and concordantly identified in 3 patients that were each analysed in three independent experiments for the detection of mutations by 454 amplicon ultra deep sequencing (A) along the amplicon and (B) at *KRAS* and *NRAS* mutation hot spot loci only. The lines represent the 90% cross validation percentage for the detected variants.

A

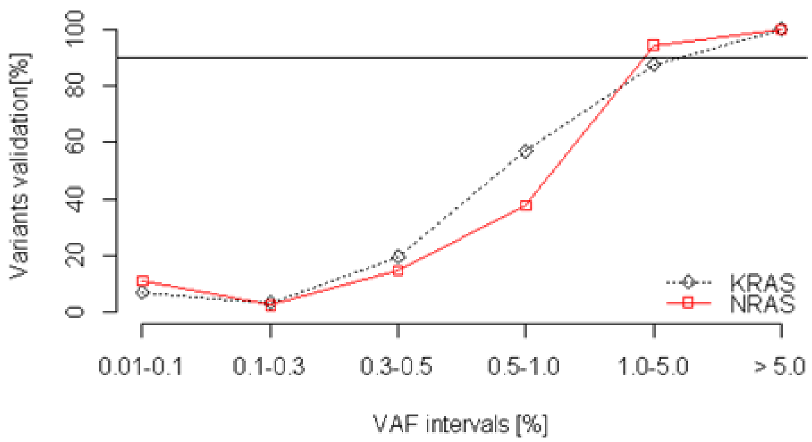

B

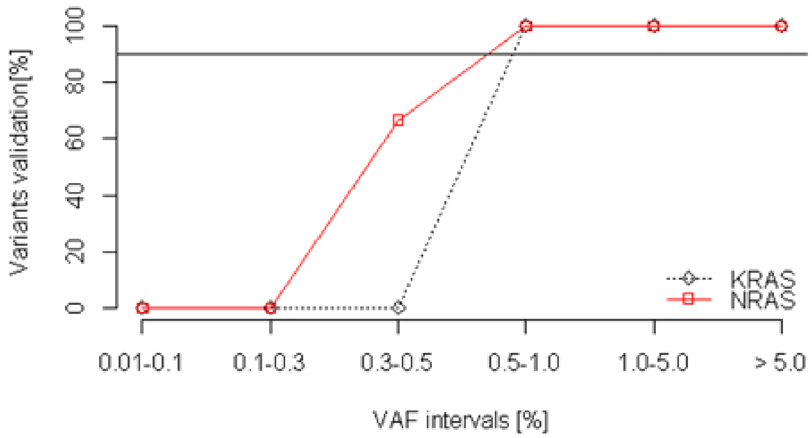

Supplementary Figure 2

The event free survival (EFS) curves of RAS^{mut} and RAS^{wt} patients are shown. P value according to Kaplan Mayer; standard error in brackets.

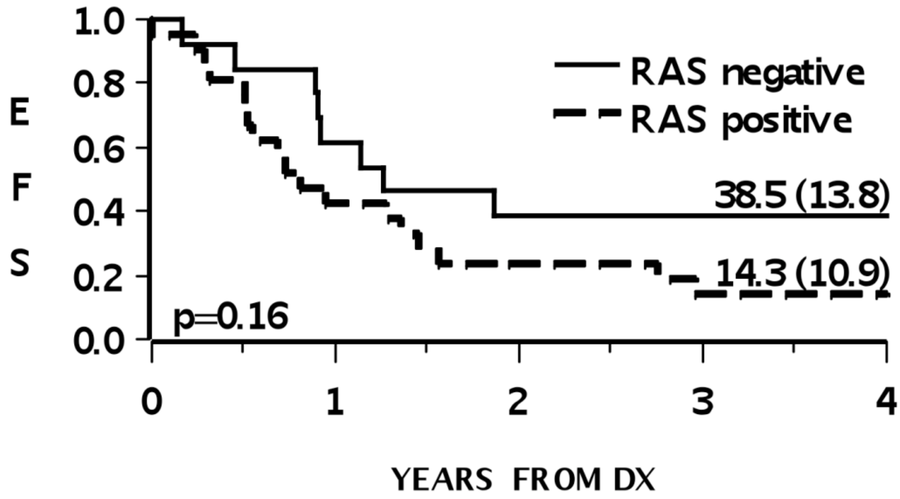

Supplementary Figure 3

Plots showing the *RAS* clones VAF during disease progression in the 14/17 mutated samples. D: diagnosis; R: relapse; 1stR: first relapse; 2ndR: second relapse; C: control time point; VAF: variant allele frequency; Pt: patient identification number.

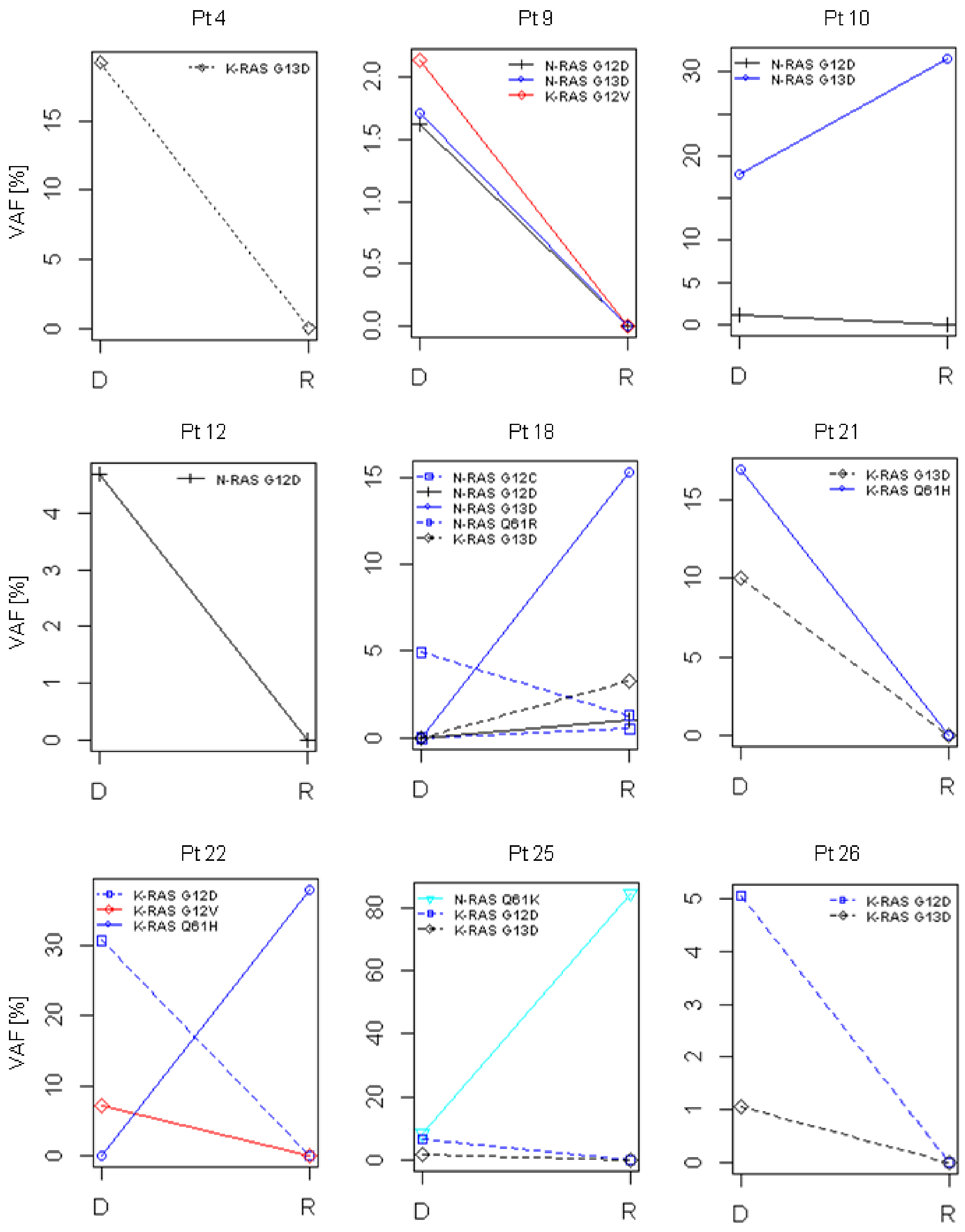

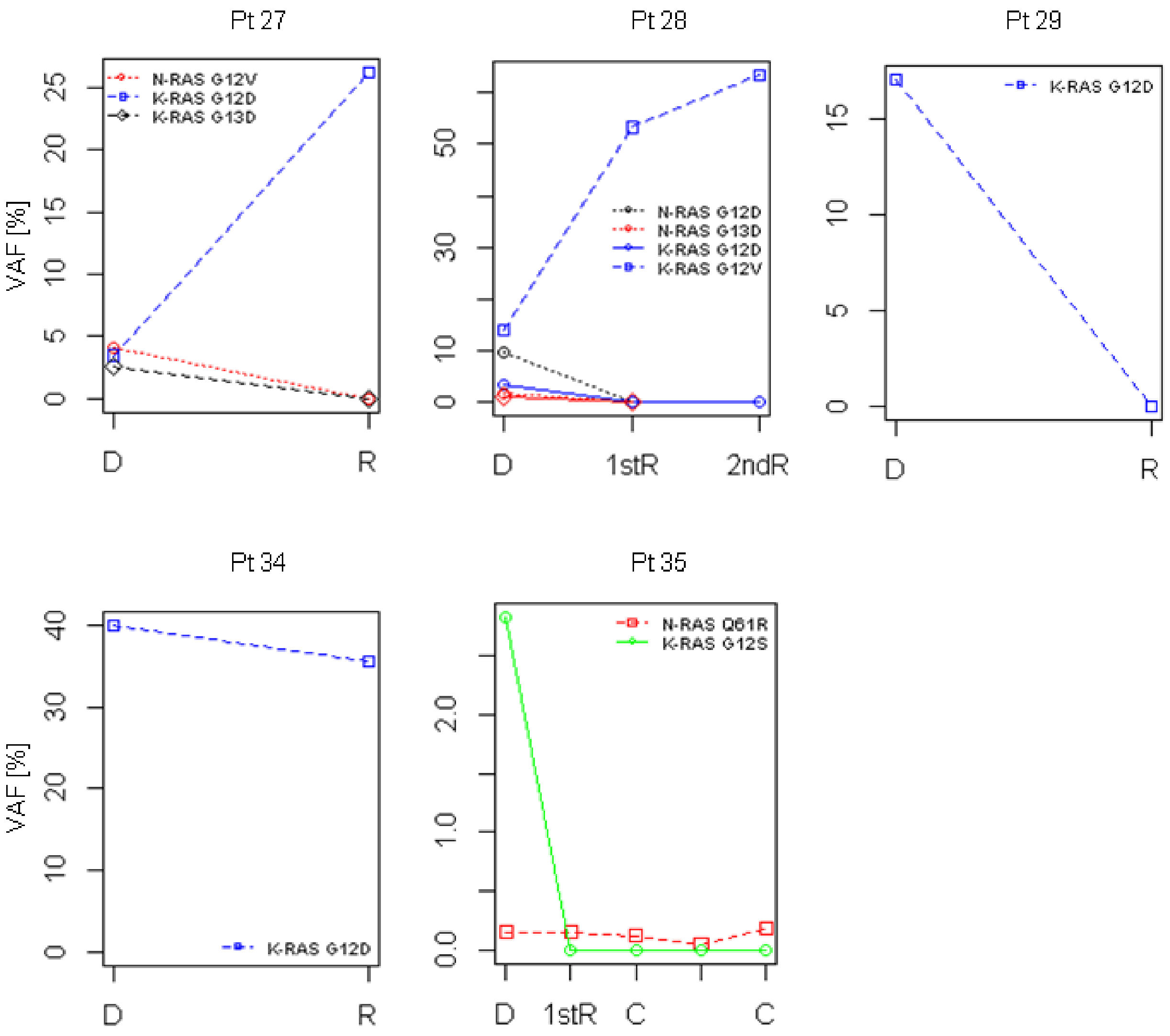
Pt 27
VAF [%]
N-RAS G12V
K-RAS G12D
K-RAS G13D
D
R
Pt 28
N-RAS G12D
N-RAS G13D
K-RAS G12D
K-RAS G12V
D
1stR
2ndR
Pt 29
K-RAS G12D
D
R
Pt 34
VAF [%]
K-RAS G12D
D
R
Pt 35
N-RAS Q61R
K-RAS G12S
D
1stR
C
C

Supplementary Figure 4

Gene set enrichment analysis (GSEA) comparing the 11 RAS^{mut} and the 5 RAS^{wt} samples independently of the *HOXA/IRX1* different gene expression level. The top 50 ranking genes differentially expressed between the two RAS phenotypes are shown in the heatmap. Red: up-regulation; blue: down-regulation.

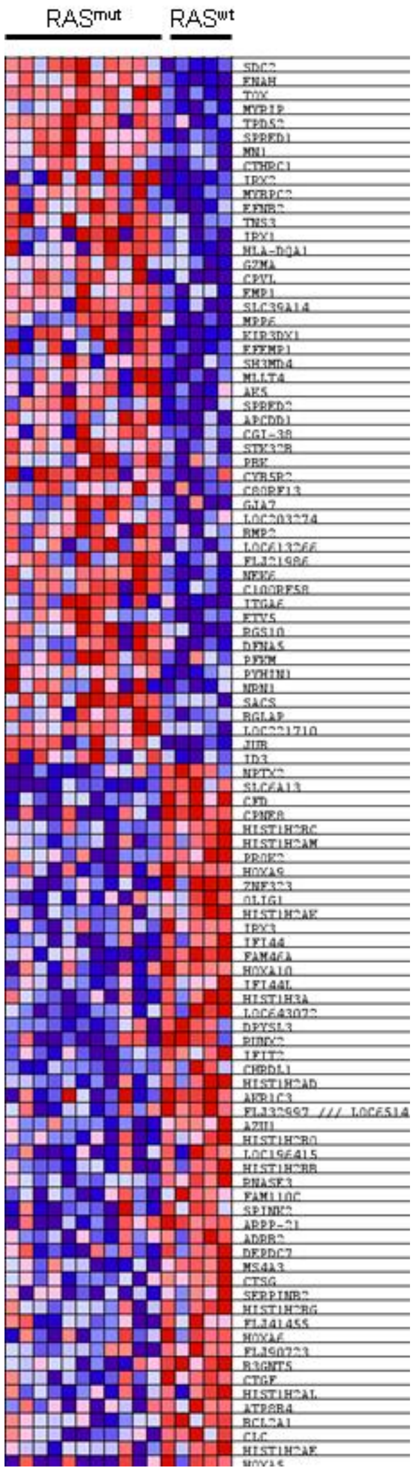

Supplementary Figure 5

Bar plot showing the single variant allele frequency (VAF) for all *KRAS* and *NRAS* hot spot mutations (N=49) detected in exons 2 and 3 of mutated MLL-AF4+ patients at diagnosis (N=23) with the 454 ROCHE GS Junior platform. The solid line represents the 20% threshold for Sanger-based sequencing; the horizontal dashed line is set to 10% and represents the lower detection limit of Sanger sequencing.

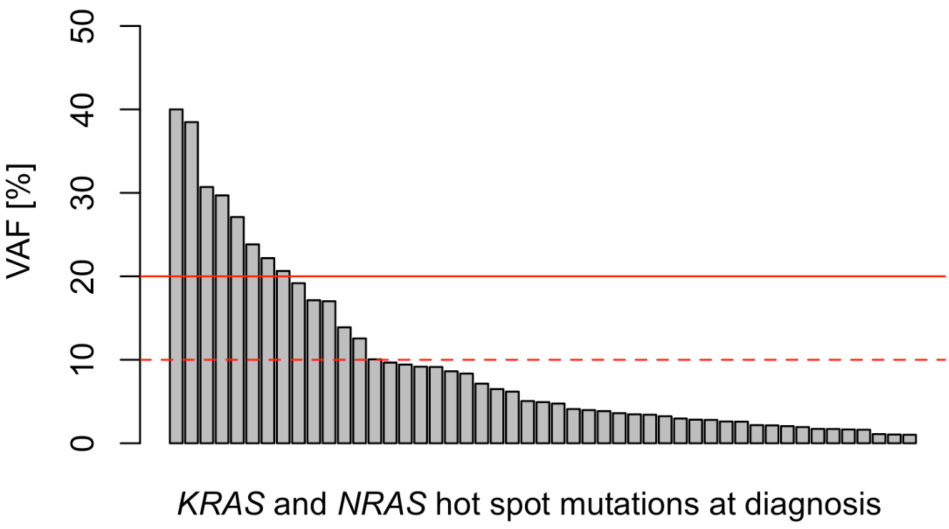

Supplementary Figure 6

Spearman correlation analysis between *FLT3* log2 expression values (probe 206674_at) and the total VAF (%) in 11 RAS^{mut} samples with available gene expression data at diagnosis (A). *FLT3* (probe 206674_at) log2 expression in the RAS^{mut} (N=11) and RAS^{wt} (N=4) patients at diagnosis (B); mean values ± S.D. are shown; p-value was calculated using the Mann-Whitney test.

A

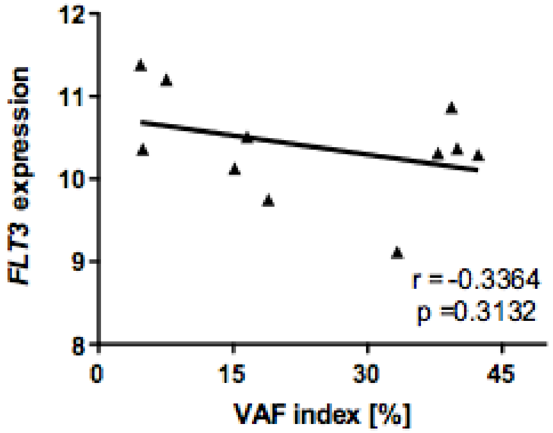

B

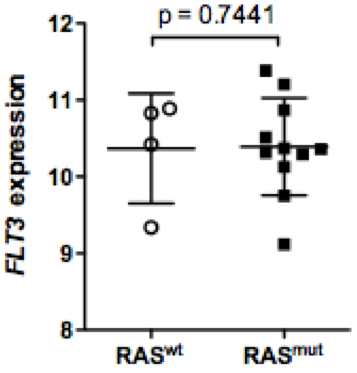

Supplementary Figure 7

Characterization of the newly established BCP-MLL-AF4 leukaemia cell line. **(A)** Sanger sequencing showing the presence of the *NRAS* Q61K mutation. **(B)** FISH analysis using a dual fusion probe for the detection of the *MLL-AF4* fusion resulting in two yellow fusion signals.

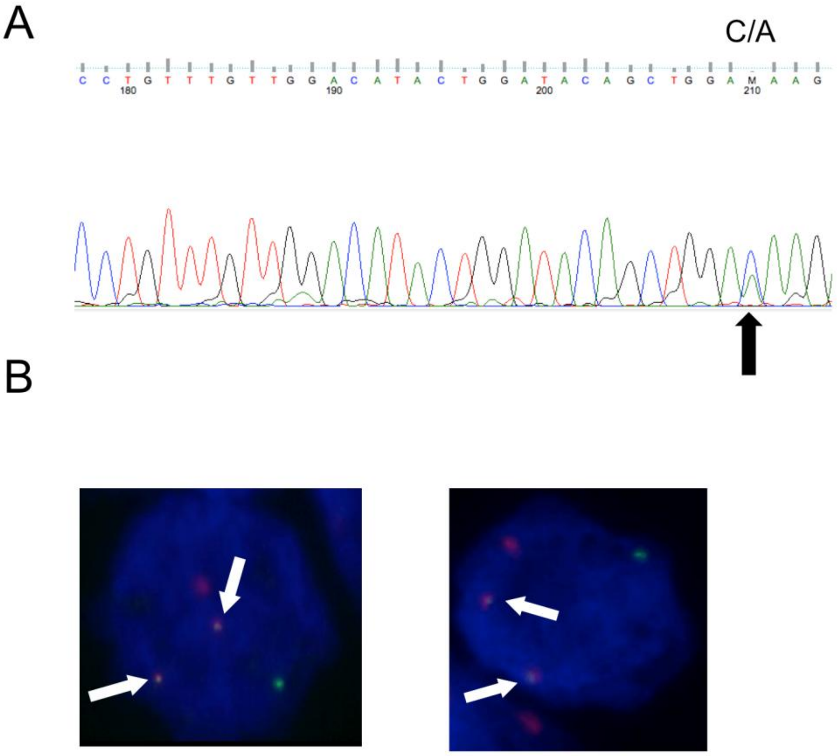

Supplement: Supplementary Information [file srep34449-s1.pdf]
